# Lymphomatosis cerebri: a rare variant of primary central nervous system lymphoma and MR imaging features

**DOI:** 10.1186/s40644-017-0128-2

**Published:** 2017-10-05

**Authors:** Hui Yu, Bo Gao, Jing Liu, Yong-Cheng Yu, Mark S. Shiroishi, Ming-Ming Huang, Wen-Xiu Yang, Zhi-Zhong Guan

**Affiliations:** 1 Department of Radiology, Affiliated Hospital of Guizhou Medical University, Guiyang, 550004 People’s Republic of China; 2 Department of Radiology, Yantai Yuhuangding Hospital, Yantai, 264000 Shandong People’s Republic of China; 3 Department of Neurology, the second affiliated Hospital of Guizhou Medical University, Kaili, 556000 People’s Republic of China; 4 Department of Radiology,Keck School of Medicine, University of Southern California, Los Angeles, CA USA; 5 Department of Pathology, Affiliated Hospital of Guizhou Medical University, Guiyang, 550004 People’s Republic of China

**Keywords:** Lymphomatosis cerebri, Primary central nervous system lymphoma, Magnetic resonance imaging, Diffusion weighted imaging, Magnetic resonance spectroscopy

## Abstract

**Background:**

Lymphomatosis cerebri (LC) is a rare variant of primary central nervous system lymphoma (PCNSL), characterized by diffuse infiltration without the formation of a discrete mass. The diagnosis of LC is a challenge because the imaging findings are atypical for lymphoma. The purpose of present study is to investigate MRI characteristics and clinical features of LC and potentially facilitate an early and accurate diagnosis of this often-missed disease.

**Methods:**

Seven patients (average 44 years, 19–58 years) with LC proved basing on MRI and histology were retrospectively reviewed the clinical data and cerebral MR imaging findings.

**Results:**

The common presenting symptoms were cognitive decline, behavioral disturbance, gait disturbance. All patients had both deep and lobar lesion distribution, and two of them had infratentorial involvement. Lack of contrast enhancement and subtle patchy enhanced pattern were observed in two and three patients, respectively. The remaining two patients presented multiple patchy enhancement. Most of the lesions were slightly hyperintense to normal brain on DWI as well as hyperintense on ADC maps. Three patients presented a pattern of marked decrease of NAA/Cr, increase of Cho/Cr, and two of the three cases showed increased Lip/Cr and Lac/Cr on MRS.

**Conclusions:**

We conclude that diffuse bilateral lesions especially in deep and lobar region including white and gray matter, without enhancement or with patchy enhancement, marked decrease of NAA/Cr and increase of Cho/Cr, and increased Lip/Cr and Lac/Cr are suggestive of LC. Prompt recognition of these imaging patterns may lead to early diagnosis of LC and brain biopsy with improved prognosis.

## Background

Lymphomatosis cerebri (LC) is a rare variant of primary central nervous system lymphoma (PCNSL), characterized by diffuse infiltration without the formation of a discrete mass and with little contrast enhancement [1], appearing similar to gliomatosis cerebri [2]. This uncommon entity is a diagnostic challenge because the clinical presentation is subacute and the imaging findings are very different from other PCNSLs which typically presents as single or multiple T2-hyperintense, nodular contrast-enhancing mass lesions on MRI [1]. Central nervous system infections, inflammatory, toxic, and metabolic disorders can also mimic the radiologic features of LC [3, 4]. Therefore, early and accurate diagnosis of LC could be crucial for its appropriate treatment choices [5]. Besides traditional MRI, diffusion weighted imaging (DWI) and magnetic resonance spectroscopy (MRS) can also be informative and useful for diagnosis of this rare disease. Neuroimaging studies of LC are rare, and the characteristics of MRI findings remain to be fully elucidated. This study was designed to retrospectively analyze the MRI and clinical manifestations of 7 patients with histopathologically confirmed LC. This summary of the MRI features of LC may facilitate early diagnosis and intervention of this often-missed disease.

## Case presentation

### Patients and methods

We retrospectively reviewed the clinical data and cerebral MR imaging of 7 patients (from January 2012 to December 2016)who were diagnosed basing on the following criteria: (i) presence of diffuse lesions in the brain MRI without contrast enhancement or with patchy contrast enhancement and (ii) histology revealing lymphoma. Patients with concurrent systemic lymphoma and intravascular lymphoma were excluded. All the patients underwent stereotactic brain biopsy and were diagnosed with lymphoma by two experienced neuropathologists (each with 7 and 15 years of experience, respectively) according to hematoxylin-eosin (H&E) staining and immunohistochemical examinations. All patients provided informed consent for MRI examination and for the use of personal data.

MRIs of three patients were performed on 3.0 Tesla Philips Achieva(Netherlands) scanner, three other cases were imaged on a 1.5 Tesla Siemens Magnetom Avanto(Germany), one patient was imaged on 1.5 Tesla GE(America). All patients had T2-weighted fluid attenuated inversion recovery(FLAIR), T2- and T1-weighted images. Post-contrast T1-weighted images were available for all patients after intravenous administration of 0.1 mmol/kg gadopentetate dimeglumine(Beilu Inc.). DWI was available in 5 patients, while MRS was available in 3 patients. The DWI was obtained using B-values of 0, 1000 s/mm^2^. The mean ADC values in the lesions and in normal-appearing white matter were measured by two experienced neuroradiologists (each with 7 and 15 years of experience, respectively). MR spectroscopy was performed with a intermediate echo time (135 ms or 40 ms) as multi-voxel or single-voxel 2D exam encompassing the lesion. For MRS, the major metabolites(Choline (Cho), N-acetylaspartate (NAA) and Creatine (Cr)) were determined. Also, the presence of lipids(Lip) and lactate(Lac) peaks was determined. For each spectrum, the peak height of total creatine (Cr) was used as the internal reference to quantify other metabolites.

The two neuroradiologists, who were blinded to patient data, reviewed and analyzed the cerebral MRI in consensus. All scans were reviewed noting lesions location in the brain, patterns of contrast enhancement, the signal of DWI and corresponding ADC map, and MRS metabolic patterns. The lesions distribution were classified into deep, lobar, and infratentorial categories(Type I,II and III respectively). Deep regions included the basal ganglia, thalamus, internal capsule, external capsule, corpus callosum, and deep and periventricular white matter (DPWM); lobar regions included cortical gray matter and subcortical regions(including lateral white matter adjacent to DPWM); infratentorial regions included the brainstem and cerebellum. DPWM was defined as white matter adjacent to or within approximately 10 mm of the lateral ventricular margin. Contrast enhancement was defined as patchy when a minimally or moderately heterogeneous, not well-defined area of contrast enhancement was present, regardless of size. The criterion for diffusion restriction lesions was hyperintense areas on DWI with corresponding hypointensity on the ADC map. Disagreements were resolved by consensus.

## Results

Our case series was composed of 3 men and 4 women, ranging from 19 to 58 years of age (average year is 44 and medium year is 49). Presenting symptoms were cognitive decline in 4, behavioral disturbance in 2, gait disturbance in 2, coma in 1 and altered level of consciousness in 1 patient. Five patients were diagnosed with diffuse large B-cell lymphoma and two patients had T-cell lymphoma. One patient was treated with corticosteroids and died 1 month after the biopsy. After courses of chemotherapy in remaining 6 patients, there was some neurological and radiological improvement in four cases, but one patient died 9 months after the biopsy and another one patient appeared a mass lesion in parietal lobe after six courses of chemotherapy.

**Fig. 1:**
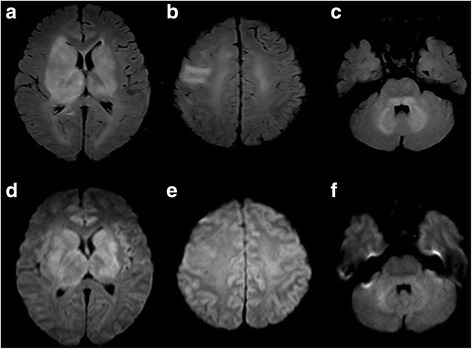
Nineteen-year-old man with lymphomatosis cerebri. **a**, **b** and **c**, Axial T2-weighted-FLAIR with STIR show the lesions distribution of type I, II and III respectively, which involved bilateral superior cerebellar peduncles, dentate nucleus of cerebellum, basal ganglia, internal capsule, thalamus and right frontal lobes. **d**, **e** and **f**, DWI images show slight hyperintensity in the lesions, but corresponding ADC maps (not shown) indicate no water of restriction

**Fig. 2:**
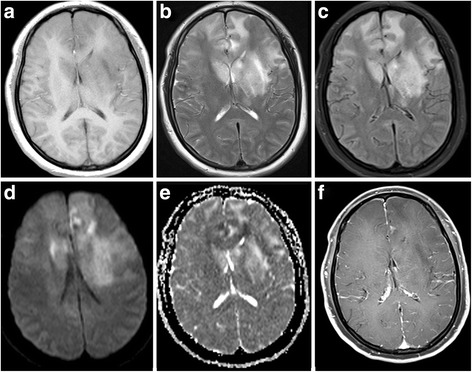
Forty five-year-old woman with lymphomatosis cerebri. **a**, **b** and **c**, Axial T1WI, T2WI and T2-weighted-FLAIR show the distribution of type I and II lesions, which involved bilateral frontal lobes and basal ganglia, left thalamus, and genu of corpus callosum. **d** and **e** *,* DWI shows slight hyperintensityand corresponding low ADC in portions of the genu of corpus callosum and left frontal lobe. **f** *,* Post-contrast T1WI shows subtle patchy contrast enhancement in the left frontal lobe

**Fig. 3:**
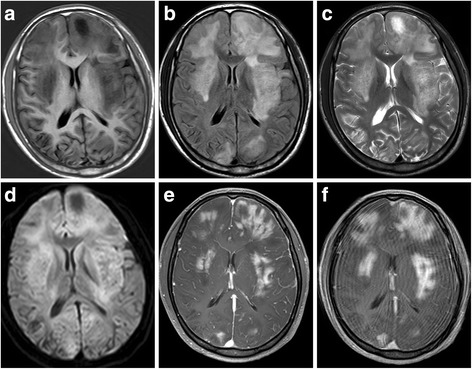
Twenty one-year-old man with lymphomatosis cerebri. **a**, **b** and **c**, Axial T1WI,T2-weighted FLAIR and T2WI show the distribution of type I and II lesions, which involved bilateral frontal lobes, occipital lobes, basal ganglia and insula. **d** DWI shows slight hyperintensity in the lesions, which were also slightly hyperintense on ADC map(not shown). **e** Post-contrast T1WI shows multiple patchy contrast enhancement in the lesions. **f**, Delayed scanning of post-contrast T1WI shows extended enhancement range of the lesions

**Fig. 4:**
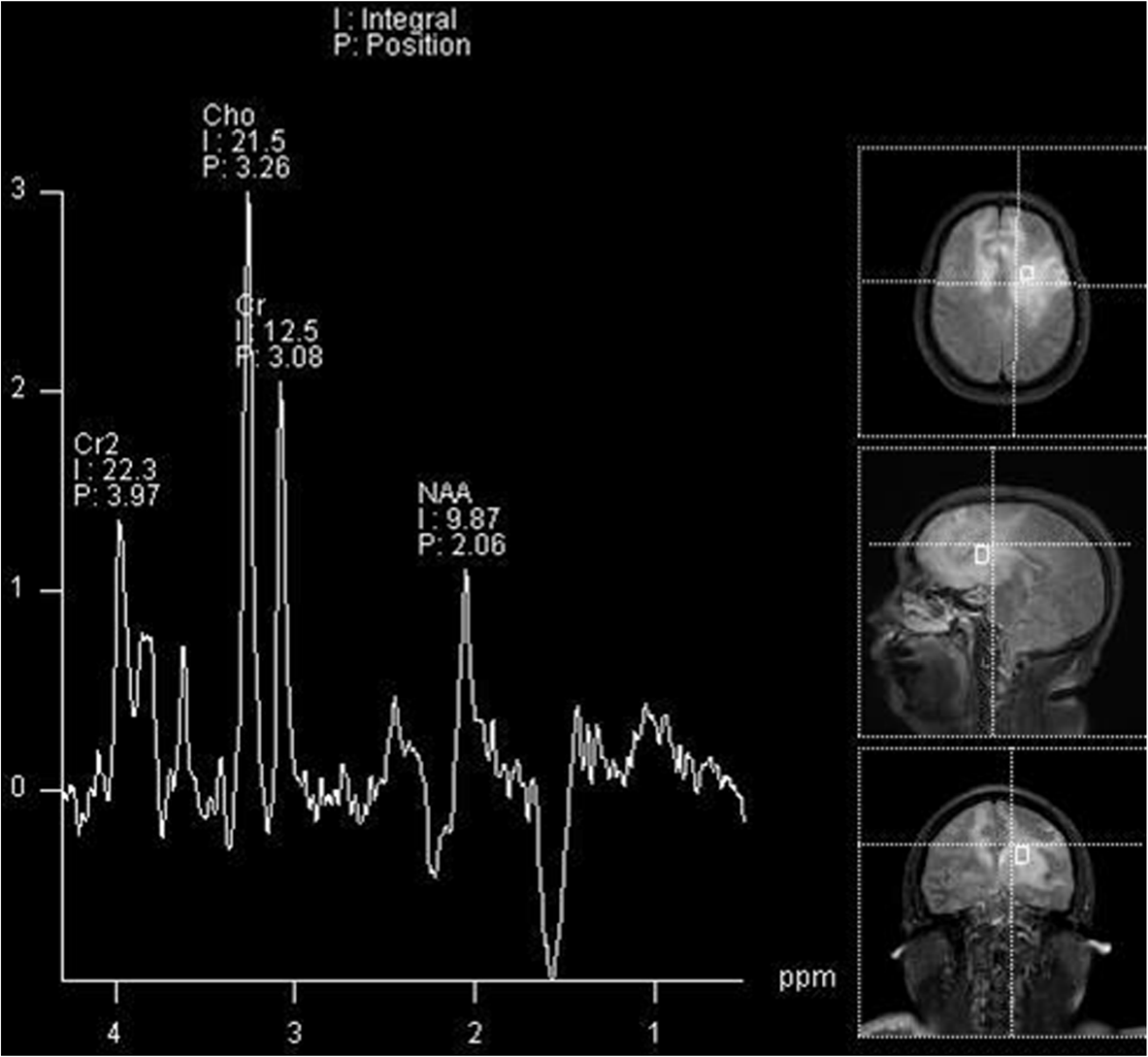
The same case shown in Fig.2. Multiple-voxel spectra are acquired with a point-resolved spectroscopy (PRESS) sequence, TR 1500 ms, TE 135 ms, NSA 128. MRS shows elevation of Cho/Cr and marked reduction of NAA/Cr in the lesion. In addition, there is an inverted Lac peak

**Fig. 5:**
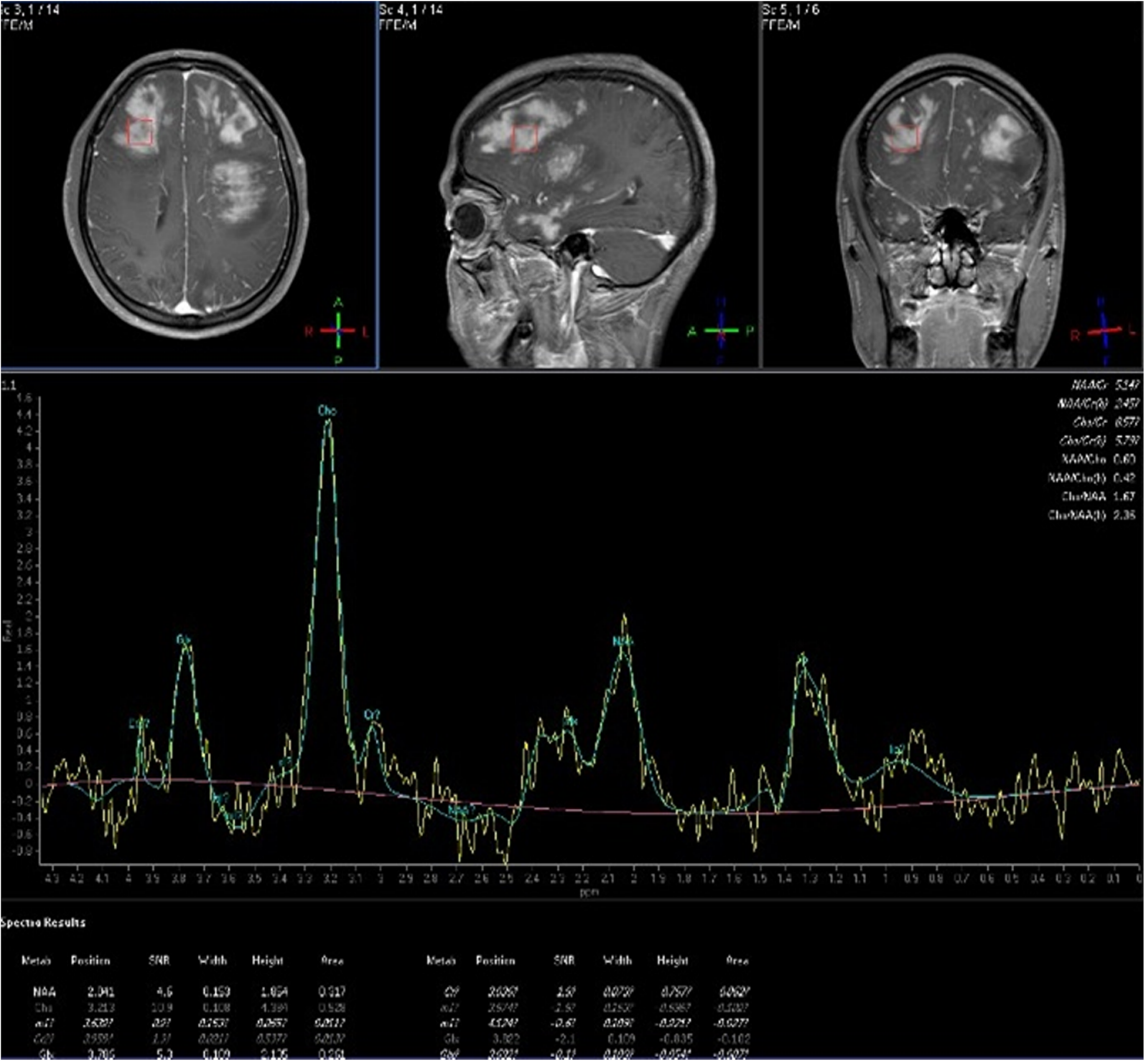
The same case shown in Fig.3. Single-voxel spectra are acquired with a point-resolved spectroscopy (PRESS) sequence, TR 2000 ms, TE 40 ms, NSA 128. MRS shows elevation of Cho/Cr and marked reduction of NAA/Cr in the lesion. In addition, there is a large Lip peak

**Fig. 6:**
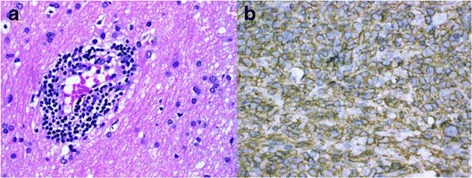
**a** Brain biopsy specimen from the case 1 shows dispersed round neoplastic cells spreading along the white matter tracts without causing tissue destruction or mass formation and are occasionally clustered around blood vessels (H&E × 400). **b** Immunohistochemistry shows that the atypical cells were positive for CD20, a marker for **b** cells

Patient demographics, cerebral MRI findings and pathological types are summarized in Table 1. Representative FLAIR, pre-contrast T1WI and post-contrast T1WI, available DWI and ADC maps, available MRS are shown in Figs. 1, 2, 3, 4 and 5. All patients had both deep and lobar lesion distribution, and two of them had infratentorial involvement. Lack of contrast enhancement and subtle patchy enhanced pattern were observed in two and three patients, respectively. The remaining two patients presented multiple patchy enhancement. Most of the lesions were slightly hyperintense to normal brain on DWI as well as hyperintense on ADC maps, which were consistent with the increasing diffusivity. Two cases showed increased peak of Lip and Lac on MRS. The pathology was characterized by dispersed round neoplastic cells spreading along the white matter tracts without causing tissue destruction or mass formation and was occasionally clustered around blood vessels (Fig. 6 A). Five cases were strongly labeled CD20, a B cell marker (Fig. 6 B) and two cases were positive for CD3, consistent with T-cell lymphoma.

**Table 1:** MRI features of the LC cases and histological diagnosis

Case	Sex/Age (years)	Types of lesions distribution			Enhancement	DWI	1H–MRS	Pathological type
		I	II	III				
1	M/55	“+”	“+”	“+”	Multiple patchy	Slightly hyperintense		B
2	F/49	“+”	“+”		Partial patchy	NE		B
3	F/45	“+”	“+”		Partial patchy	Slightly hyperintense	Cho/Cr ↑ NAA/Cr↓ Lac/Cr ↑	B
4	F/57	“+”	“+”		Partial patchy	Slightly hyperintense		B
5	M/27	“+”	“+”		Multiple patchy	Slightly hyperintense	Cho/Cr ↑ NAA/Cr↓ Lip/Cr ↑	T
6	M/19	“+”	“+”	“+”	No	Slightly hyperintense	Cho/Cr ↑ NAA/Cr↓	T
7	F/58	“+”	“+”		No	NE		B

*LC* lymphomatosis cerebri, *NE* not evaluated, *DWI* diffusion-weighted imaging, *MRS* magnetic resonance spectroscopy, *Cho* choline, *Lip* Lipid, *Lac* lactate, *NAA N*-acetylaspartate, *Cr* creatine, *B* diffuse large B-cell lymphoma, *T*, T-cell lymphoma

## Discussion

About 90% of PCNSL’s are diffuse large B-cell lymphomas and only 5% constitute low-grade B-cell type lymphoma, including mucosa-associated lymphoid tissue type and T-cell type lymphoma [6]. LC is a rare type of CNS lymphoma characterized by lymphoma cells diffusely infiltrating the brain parenchyma without forming a mass or distorting the cerebral architecture [2]. MR imaging typically reveals diffuse white matter disease variably involving bilateral cerebral hemispheres, periventricular region, basal ganglia, thalami, or the brainstem [1]. With the aim of improving knowledge of this entity, we documented the clinical features and brain MR imaging findings from 7 patients with LC and analyzed the abnormal findings of multiparametric MRI focusing on: 1) distribution of lesions on conventional MRI, 2) patterns of contrast enhancement, 3) signal of DWI, and 4) metabolite changes on MRS.

### Clinical features

Patients with LC may have variable clinical symptoms such as gait disturbance, focal weakness, decline of cognitive function, memory disturbance, personality changes, dementia, anorexia, orthostatic hypotension, paraparesis and weight loss [3]. Of these, the most common presenting symptoms are cognitive decline(59.5%), gait disturbances(54.8%) and behavioral changes(50%) [5]. Similar to most reported cases presented with rapidly progressing dementia accompanied by extensive leukoencephalopathy on MRI [2, 7, 8], four of our cases were typical of previously described LC cases. But the clinical presentation of these patients were easily mistaken for other, more common, conditions such as infectious, inflammatory, vascular, toxic, or neurodegenerative etiologies that can cause white-matter injury. Our cases suggested that it is important for clinicians to be aware of this LC form of PCNSL and LC should be added to the differential diagnosis of cognitive decline.

### Distribution of lesions

It is noteworthy that all the patients had bilateral hemispheric lesions on MRI. Conventional MRI without contrast enhancement showed extensive, diffuse hyperintense lesions involving bilateral cerebral hemispheres on both T2-weighted FLAIR sequences. Case 1 and 6 of our series had concurrent infratentorial and supratentorial infiltration. The remaining patients presented with isolated supratentorial infiltration. The most common regions involved were the white matter of both hemispheres, in the frontal and deep periventricular regions including corpus callosum, and the lesions extended into the gray matter such as basal ganglia,thalamus and cortex. We classified these lesions by distribution into deep, lobar, and infratentorial categories as TypeI,II and III, respectively. The two most common lesion distribution in LC included those in the deep and lobar categories regardless if the lesions involved gray or white matter. The lesions of deep brain regions type were always bilateral and incompletely symmetrical distribution in LC, but the lesions of lobar type were always unilateral. Lymphomatous cells have a tendency to traffic in rows between white matter fibers rather than expanding diffusely [9]. The diffuse findings can be explained by the theory that lymphoma has to be considered as a whole brain disease even when it presents with a cohesive mass [10].

Given the multifocal distribution of the lesions in white matter, the main differential considerations were Binswanger’s disease(subcortical ischemic vascular dementia), infectious leukoencephalitis, toxic encephalopathy, or neoplasms such as gliomatosis cerebri [2, 11–13]. However, diffuse involvement of both hemispheres and incompletely symmetrical distribution, including involved white matter and deep gray matter simultaneously, distinguish LC from other entities considered in the differential diagnosis.

### Patterns of contrast enhancement

Most patients of our series showed patchy contrast enhancement. Bakshi et al. [2] defined cases of LC as diffuse white matter infiltration without the formation of discrete mass lesions and with little contrast enhancement. Rollinset al. [12]reviewed the pathological findings of LC. In LC, there is a diffuse pattern of brain infiltration coupled with the associated perivascular cuffing by both lymphoma cells and non-neoplastic lymphocytes that can mimic an encephalitic pattern [2]. The common reason for a lack of contrast enhancement on MRI is assumed to be an intact blood-brain barrier (BBB), or that significant BBB disruption by lymphoma cells is not yet produced [14]. However, subtle or patchy contrast enhancement has been described in some cases [6, 7]. In these cases, biopsy revealed tumor cells that induced subtle contrast enhancement distributed throughout the white matter; the atypical cells were neither cohesive nor did they form a mass [3]. Subtle patchy contrast enhancement was found in 3 of our 7 patients and 2 patients showed multiple patchy. Our Case 5 showed more substantial contrast enhancement in both cerebral hemispheres, which was misdiagnosed with encephalitis based on the initial MRI and improved after steroid treatment. Histopathological analysis of this patient revealed severe tumor cell infiltration with small round lymphatic cells cuffing and destroying microvasculature, which is consistent with the imaging finding of multi-focal patchy contrast enhancement due to BBB disruption. Delayed scanning post-contrast T1WI was 20 min after the initial scan and showed marked contrast enhancement (Figure 3). Other reports [4, 15] have reported that contrast enhancement patterns can change in LC patients on follow-up MRI. A systematic review LC patients found that 26.6% of those without contrast enhancement on the initial MRI and 16.6% of those who showed patchy contrast enhancement eventually developed nodular contrast enhancing lesions at follow-up imaging [5]. The transformation from a non-enhancing to enhancing lesion reflects the eventual disruption of the BBB [16]; this is likely a late event that is due to factors at the cellular level [17]. Although the reason for this transformation remains unclear, we speculate that LC without contrast enhancement might be an early-stage appearance of this specific type of PCNSL with diffuse infiltrating neoplastic cells.

### Diffusion weighted imaging

DWI reflects the motion of intra- and extra-cellular water. It is helpful in distinguishing between PCNSL and other tumors and tumor-mimicking lesions [18]. Highly cellular tumors, like central nervous system(CNS) lymphoma, generally present as single or multiple contrast-enhancing mass lesions on MRI scans, with hyperintensity on DWI and hypointensity on ADC maps [19]. Decreased ADC value suggests increased cellularity [20].We observed subjective diffusion restriction in portions of the lesions in two cases, suggesting high cellularity in these portions of the lesions. But most of the lesions were slightly hyperintense to normal brain on DWI and hyperintense on ADC maps, consistent with increased diffusivity. The hyperintensity on DWI and hyperintensity on ADC map may have reflect diffuse cerebral infiltration of non-cohesive malignant lymphoid cells and T2 shine-through effect. Histopathological analysis revealed blastic lymphocytic cells with large pleomorphic nuclei and distinct nucleoli diffusely infiltrating the parenchyma. These lymphocytes showed the typical angiocentric infiltration pattern, and tumor cells invaded the neural parenchyma with a diffuse growth pattern from these perivascular cuffs. Although DWI was not suggestive of LC in our cases, the variation of DWI signal intensity may be a reflection of the variation of tumor cellular density. However, the slight DWI hyperintensity is difficult to differentiate from gliomatosis cerebri, infectious leukoencephalitis, and toxic encephalopathy.

### MR spectroscopy(MRS)

MRS allows for the semiquantitative in-vivo evaluation of metabolites, such as NAA, Cho, Cr, Lac, and Lip. In PCNSL,MRS has demonstrated elevated Lip and Lac peaks, high Cho/Cr ratios, decreased NAA levels and high Cho/NAA ratios [21, 22]. PCNSL grows rapidly and behaves similar to other high-grade brain tumors with evidence of high cell membrane turnover on MRS(high Cho peak), neuronal damage (decreased NAA levels), and anaerobiosis (high lactate levels) [18, 19, 22].These findings are similar to those for high-grade gliomas and metastases; however, MRS is useful to suggest PCNSL because lipids were found to be useful to discriminate between PCNSL and glioblastoma/metastasis at short TE [23].In our three LC patients, MRS (at TE 135 ms or 40 ms) consistently presented a pattern of marked decrease of NAA/Cr, increase of Cho/Cr, which is suggestive of malignant neoplastic disease. Two patients showed increased Lip/Cr and Lac/Cr. The presence of this MRS pattern may help in the differential diagnosis of brain non-neoplastic diseases [20]. We determined that MRS is potentially useful to reinforce the suspicion of LC when bilateral hemispheric lesions were found on an MRI exam. Although LC is a relatively rare type of PCNSL, establishing suspicion of LC by imaging could be a pivotal step in determining management strategies for patients, as it would result in a consideration of biopsy before initiation of treatment with steroids [23].

### Limitations

There are several limitations in this study. First, LC is rare and the cases came from multiple institutions. As a result, we had a small sample size and heterogeneity of the dataset where DWI and MRS were not available in all cases; variable imaging sequences were performed in each case. Other advanced imaging techniques were also not available for these challenging cases, including MR perfusion and diffusion tensor imaging (DTI). Perfusion MRI can improve the diagnostic accuracy of PCNSL, particularly when the brain parenchyma is affected [24]. DTI takes advantage of highly ordered white matter fibers, and the FA values for PCNSL can help in the differentiation of glioblastoma [25].

## Conclusion

In conclusion, it is important for clinicians and radiologists to be aware of the LC form of PCNSL. Diffuse bilateral lesions especially in deep and lobar region including white and gray matter, without enhancement or with patchy enhancement, marked decrease of NAA/Cr and increase of Cho/Cr, and increased Lip/Cr and Lac/Cr are suggestive of LC. Prompt recognition of these imaging patterns may lead to early diagnosis of LC and brain biopsy with improved prognosis.

## Abbreviations

ADC: Apparent diffusion coefficient
BBB: Blood-brain barrier
Cho: Choline
CNS: Central nervous system
Cr: Creatine
DPWM: Deep and periventricular white matter
DTI: Diffusion tensor imaging
DWI: Diffusion-weighted imaging
FLAIR: Fluid attenuated inversion recovery
HE: Hematoxylin-eosin staining
Lac: Lactate
LC: Lymphomatosis cerebri
Lip: Lipid
MRS: Magnetic resonance spectroscopy
NAA: N-acetylaspartate
PCNSL: Primary central nervous system lymphoma

## References

[1] Hatanpaa KJ, Fuda F, Koduru P, Young K, Lega B, Chen W (2015). Lymphomatosis Cerebri: A Diagnostic Challenge. JAMA Neurol.

[2] Bakshi R, Mazziotta JC, Mischel PS, Jahan R, Seligson DB, Vinters HV (1999). Lymphomatosis cerebri presenting as a rapidly progressive dementia: clinical, neuroimaging and pathologic findings. Dement Geriatr Cogn Disord.

[3] Kitai R, Hashimoto N, Yamate K, Ikawa M, Yoneda M, Nakajima T, Arishima H, Takeuchi H, Sato K, Kikuta K (2012). Lymphomatosis cerebri: clinical characteristics, neuroimaging, and pathological findings. Brain Tumor Pathol.

[4] Lewerenz J, Ding XQ, Matschke J, Schnabel C, Emami P, von Borczyskowski D, Buchert R, Krieger T, de Wit M, Munchau A. Dementia and leukoencephalopathy due to lymphomatosis cerebri. BMJ Case Rep. 2009;2009 10.1136/bcr.08.2008.0752. 10.1136/bcr.08.2008.0752 PMC3028137 21686648

[5] Izquierdo C, Velasco R, Vidal N, Sanchez JJ, Argyriou AA, Besora S, Graus F, Bruna J (2016). Lymphomatosis cerebri: a rare form of primary central nervous system lymphoma. Analysis of 7 cases and systematic review of the literature. Neuro-Oncology.

[6] Sugino T, Mikami T, Akiyama Y, Wanibuchi M, Hasegawa T, Mikuni N (2013). Primary central nervous system anaplastic large-cell lymphoma mimicking lymphomatosis cerebri. Brain Tumor Pathol.

[7] Raz E, Tinelli E, Antonelli M, Canevelli M, Fiorelli M, Bozzao L, Di Piero V, Caramia F (2011). MRI findings in lymphomatosis cerebri: description of a case and revision of the literature. J Neuroimaging.

[8] Weaver JD, Vinters HV, Koretz B, Xiong Z, Mischel P, Kado D (2007). Lymphomatosis cerebri presenting as rapidly progressive dementia. Neurologist.

[9] Vital A, Sibon I (2007). A 64-year-old woman with progressive dementia and leukoencephalopathy. Brain Pathol.

[10] Lai R, Rosenblum MK, DeAngelis LM (2002). Primary CNS lymphoma: a whole-brain disease?. Neurology.

[11] Filley CM, Kleinschmidt-DeMasters BK (2001). Toxic leukoencephalopathy. N Engl J Med.

[12] Rollins KE, Kleinschmidt-DeMasters BK, Corboy JR, Damek DM, Filley CM (2005). Lymphomatosis cerebri as a cause of white matter dementia. Hum Pathol.

[13] Lewerenz J, Ding X, Matschke J, Schnabel C, Emami P, von Borczyskowski D, Buchert R, Krieger T, de Wit M, Munchau A (2007). Dementia and leukoencephalopathy due to lymphomatosis cerebri. J Neurol Neurosurg Psychiatry.

[14] Terae S, Ogata A (1996). Nonenhancing primary central nervous system lymphoma. Neuroradiology.

[15] Courtois F, Gille M, Haven F, Hantson P (2012). Lymphomatosis cerebri Presenting as a Recurrent Leukoencephalopathy. Case Rep Neurol.

[16] Samani A, Davagnanam I, Cockerell OC, Ramsay A, Patani R, Chataway J (2015). Lymphomatosis cerebri: a treatable cause of rapidly progressive dementia. J Neurol Neurosurg Psychiatry.

[17] Phan TG, O'Neill BP, Kurtin PJ (2000). Posttransplant primary CNS lymphoma. Neuro-Oncology.

[18] da Rocha AJ, Sobreira Guedes BV, da Silveira da Rocha TM, Maia Junior AC, Chiattone CS (2016). Modern techniques of magnetic resonance in the evaluation of primary central nervous system lymphoma: contributions to the diagnosis and differential diagnosis. Rev Bras Hematol Hemoter.

[19] Zacharia TT, Law M, Naidich TP, Leeds NE (2008). Central nervous system lymphoma characterization by diffusion-weighted imaging and MR spectroscopy. J Neuroimaging.

[20] Haldorsen IS, Espeland A, Larsson EM (2011). Central nervous system lymphoma: characteristic findings on traditional and advanced imaging. AJNR Am J Neuroradiol.

[21] Taillibert S, Guillevin R, Menuel C, Sanson M, Hoang-Xuan K, Chiras J, Duffau H (2008). Brain lymphoma: usefulness of the magnetic resonance spectroscopy. J Neuro-Oncol.

[22] Raizer JJ, Koutcher JA, Abrey LE, Panageas KS, DeAngelis LM, Lis E, Xu S, Zakian KL (2005). Proton magnetic resonance spectroscopy in immunocompetent patients with primary central nervous system lymphoma. J Neuro-Oncol.

[23] Mora P, Majos C, Castaner S, Sanchez JJ, Gabarros A, Muntane A, Aguilera C, Arus C (2014). **(**1)H-MRS is useful to reinforce the suspicion of primary central nervous system lymphoma prior to surgery. Eur Radiol.

[24] Lee IH, Kim ST, Kim HJ, Kim KH, Jeon P, Byun HS (2010). Analysis of perfusion weighted image of CNS lymphoma. Eur J Radiol.

[25] Toh CH, Castillo M, Wong AM, Wei KC, Wong HF, Ng SH, Wan YL (2008). Primary cerebral lymphoma and glioblastoma multiforme: differences in diffusion characteristics evaluated with diffusion tensor imaging. AJNR Am J Neuroradiol.

